# “I Can Remember Thinking, Like Almost Wishing, That the Injuries Would Have Been Worse, Because Then I Wouldn’t Be Questioned”: A Qualitative Study on Women’s Experience of Accessing Healthcare for Intimate Partner Violence-Related Brain Injury

**DOI:** 10.3390/healthcare14020165

**Published:** 2026-01-08

**Authors:** Eve M. Valera, Isha Sanghvi, Sarah Rose Sitto, Jason Chua, Altaf Saadi, Alice Theadom

**Affiliations:** 1 Department of Psychiatry, Harvard Medical School, Massachusetts General Hospital, Charlestown, MA 02129, USA; 2 Stanford University School of Medicine, Stanford, CA 94305, USA; isanghvi@stanford.edu; 3 College of Optometry, Midwestern University, Downers Grove, IL 60515, USA; ssitto@bu.edu; 4 AUT Traumatic Brain Injury Network, Faculty of Health and Environmental Sciences, Auckland University of Technology, Auckland 0627, New Zealand; jason.chua@aut.ac.nz (J.C.); alice.theadom@aut.ac.nz (A.T.); 5 Department of Neurology, Harvard Medical School, Massachusetts General Hospital, Boston, MA 02114, USA; asaadi@mgh.harvard.edu

**Keywords:** intimate partner violence, brain injury, qualitative, interviews, healthcare access

## Abstract

**Background/Objectives**: To identify the barriers and facilitators to accessing healthcare following intimate partner violence (IPV)-related brain injury (BI). **Methods**: Sixteen adult women participated in interviews about their experience of accessing healthcare following IPV-related BI. Interviews were transcribed verbatim and analyzed using the interpretative descriptive (ID) approach to identify themes and subthemes in the data. **Results**: Two themes, each with six subthemes related to healthcare seeking for IPV-related BI were identified: Theme 1—Deciding to seek and ability to access healthcare, comprising (a) severity of injury; (b) impact of injury; (c) ability to access medical services; (d) self-blame, fear, shame, and guilt; (e) contextual influences on healthcare seeking; and (f) previous negative interactions; and Theme 2—Complexity in identifying IPV-related BI, comprising (a) trauma can affect recall of events; (b) inability to distinguish IPV-related trauma or aging outcomes from BI sequelae; (c) the importance of trust in disclosure; (d) healthcare professionals need to ask the right questions and respond in the right way; (e) the complex nature of disclosure creates challenges for diagnosis; and (f) fear of being dismissed or judged. **Conclusions**: Many context-related factors influence whether women can seek treatment for IPV-related BIs. These factors need to be understood by first responders and medical professionals to improve the likelihood and speed of treatment seeking. Furthermore, challenges and fears associated with disclosure of IPV prevent women from seeking proper treatment. IPV training could be helpful in ensuring women feel safe with disclosure.

## 1. Introduction

Globally, an estimated one in three women experience physical or sexual intimate partner violence (IPV) in their lifetime [1]. Injuries can be fatal, with IPV representing a leading cause of homicide for all women globally [2]. Head and neck IPV-related injuries are particularly common, with data suggesting very high prevalence rates [3,4,5,6]. Further, single and repetitive IPV-related brain injuries, either from blunt force trauma or strangulation, are also reported to be common. For example, Valera and Berenbaum (1993) found that nearly 75% of women who experienced IPV sustained at least one IPV-related brain injury (BI) and just over half sustained repetitive IPV-related BIs [7]. Despite the scope and severity of this issue in IPV, BI research remains disproportionately focused on military and sport-related injuries.

BIs from IPV present unique challenges in both identification and treatment. For example, identification may be challenged by high levels of psychiatric comorbidity and other physical injuries that may mask BI-related symptoms. Without prompt detection and diagnosis, women experiencing IPV are likely at a higher risk for sustaining repetitive injuries with little or no recovery time in between, which, in turn, may increase risk for persistent symptoms. Women who experience IPV may also face social stigma and fear, which can complicate their ability to seek and receive healthcare. Further, neuropsychiatric outcomes, alongside the interplay of life course trauma and injury, can make determining if a brain injury has occurred more challenging [8]. As such, BI research specific to the experiences of these women is needed, rather than simply translating BI research from other groups (e.g., male athletes) to women who experience IPV-related BIs.

Currently, little is known about women’s experiences with accessing care post IPV-BIs, although there is existing literature relating to accessing care post-IPV more broadly. Women who experience IPV—particularly women of color and lower socioeconomic status—report delaying visits to medical providers to avoid disclosing IPV [9]. Factors creating hesitation to seek medical treatment or to disclose IPV range from previous negative interactions with medical professionals to fear of prosecution and/or loss of child custody [10,11]. Documented facilitators for healthcare seeking behavior include extreme injury or a mother’s concern for their child’s safety [12]. However, even then, healthcare seeking does not guarantee IPV disclosure. Studies have demonstrated that while roughly 50% of women who visit the emergency department have experienced IPV, only 5% were identified as such by healthcare professionals [13]. Less is known about healthcare seeking specifically for IPV-related BIs, which are commonly invisible, under-detected, and/or not addressed. As an example, a recent study showed that, despite a high prevalence of IPV-related BIs in a large hospital, very few referrals were made to address this issue [14]. Calls are increasingly being made to establish a greater awareness and provide resources for IPV-BI both in clinical practice and research [15,16,17]. Understanding the decision-making process about when and why women who experience IPV-related BIs seek healthcare or disclose their IPV-related BI to healthcare professionals is critical to inform intervention strategies that encourage disclosure for both IPV and BI so that women can receive timely and appropriate treatment.

We attempt to fill this gap in the literature through a qualitative study of women who have experienced IPV-related BIs. We aimed to identify the barriers and facilitators to accessing healthcare following IPV-related BI.

## 2. Materials and Methods

### 2.1. Study Design

We used a constructivist approach, applying the interpretive description (ID) method to construct women’s experiences of accessing care post IPV-BI. ID is an approach designed for application to the healthcare context and enables the generation of practical outcomes and recommendations for practice [18,19,20]. The ID approach acknowledges that people’s experiences of events include complex interactions between psychosocial and physiological factors [19]. We used the ID approach for the purpose of capturing themes and patterns and generating an interpretive description capable of informing changes to clinical understanding and practice. The ID approach enables researchers to capture an in-depth understanding of the participant perspective, while accommodating individual variation in experience, including positive or negative experiences of an identified concept [21,22]. The approach recognizes the value of a researcher’s personal and technical insight into phenomena to enable generation of insightful themes that will be useful to health and social care providers [22].

This study was approved by the Mass General Brigham Human Research Affairs Institutional Review Board (Protocol approval#: 2019P000220, approved on 25 April 2019). Verbal and written informed consent were obtained prior to conducting the interview. Participants were informed of their rights as a research participant, ability to withdraw from the study at any time, and limits of confidentiality.

### 2.2. Sample and Recruitment

From March 2023 through December 2023 participants were recruited from a larger study examining the effects of partner violence on women’s health [23]. To be eligible to enroll in the larger study, an individual was required to identify as a woman, be English-speaking, at least 18 years of age, and have experienced at least one instance of physical IPV. For the current study, women were also required to have sustained at least one IPV-related BI. As part of the larger study, BIs were assessed with two measures: the Ohio State Identification Method (OSU-ID) [24] and the Brain Injury Severity Interview (BISA) [7]. In short, the OSU-ID asks about potential trauma to the head and neck followed by loss of consciousness (LOC) or feeling dazed or experiencing a memory gap. The BISA asks about potential alterations in consciousness (AICs; e.g., LOC, feeling dazed, memory gap, seeing stars, dizziness) experienced from a partner with follow-up questions about what caused the AIC (e.g., blunt force trauma to the head, strangulation). In short, alterations in consciousness from either blunt force trauma or strangulation were considered BIs. Participants did not necessarily self-identify as having a BI, rather BIs were inferred by the PI based in the information acquired from these interviews. Participants were not informed about their BI “status” based on this assessment. More details regarding BI assessment can be found in work published on the larger samples here [23].

Eligible women who expressed interest in the study were selected to create a diverse sample of women by race, ethnicity, and brain injury experience (e.g., number of BIs, TBIs, strangulation-related BIs). Study team members called and/or emailed eligible women about the study. No women were currently in an abusive relationship, and most were at least approximately one year out of the relationship with many being decades out of the relationship. Nonetheless, information was gathered and care was taken to ensure there was no risk of re-perpetration related to the interview.

Interested women were able to ask any questions that they had about the study before a mutually convenient time was arranged to complete the interview.

### 2.3. Data Collection

Teleconference (Zoom) interviews were arranged at a mutually convenient time with women who were asked to find a private space (usually their respective homes) for the interview. Either the PI (EV) or one female research assistant (SSR) conducted the interviews. A semi-structured interview guide was used to explore women’s experiences accessing care following IPV-related BI (Supplementary Materials S1). Interviews lasted between 45 and 90 min and were audio-recorded, transcribed by Zoom, and then updated and checked for accuracy by one of the authors (IS). All reported quotes were double checked for accuracy by a research assistant in the lab. Names of people and places were removed to anonymize the data. Transcripts were uploaded to Nvivo (QSR International, Burlington, MA, USA, ver.14, 2023) for analysis. Nvivo was used to organize the data, highlight and code relevant sections of the text, or to identify quotes used in the manuscripts. Code names and descriptions were generated by the researchers.

### 2.4. Data Analysis

The anonymized interview transcripts were read several times by researchers to familiarize themselves with the data. Preliminary interpretations, and meanings in the data were noted alongside sections of the transcript to identify important issues, potential themes, or patterns. The researchers considered sections of the transcripts by asking themselves “what is the meaning behind what is being said here?”, “What is happening here?”, “How does this link to what previous participants have said, is it the same or different? If it is different, why is the experience different?” A series of initial codes were then developed to summarize the identified meanings. Two to three researchers independently coded each transcript to reduce personal bias in interpretation and to ensure analysis reflected the experiences of participants. After 11 interviews the initial code list was reviewed, refined, and re-applied to the transcripts. Researchers involved in the analysis met regularly during data collection and analysis to discuss and refine codes and to ensure a consistent approach. Throughout the research process the authors acknowledged the influence of their gender and clinical/research background and interests in IPV and BIs on the research to reduce potential biases in interpretation of the data. They utilized multiple coders with different levels of knowledge and experience with IPV and BI, maintained detailed records of the coding process, and checked codes accurately reflected the data by referring back to the quotes within each code

Data collection and analysis occurred concurrently, with initial identified concepts explored further in subsequent interviews to enable elaboration, validation, or clarification of interpretations. The codes were then refined, collapsed, or expanded to capture the diversity of experience across all transcripts. Potential links between codes were then explored, with linked codes grouped together to form themes and subthemes to capture higher level meanings. The final codes and themes were constructed to ensure they captured the breadth of participant experience and meanings that came through most strongly in participant interviews.

## 3. Results

Sixteen women (fifteen cisgender and one transgender), participated in the study. Please see Table 1 for demographic information as well as information about type and number of BIs sustained. We also note that all women sustained their most recent IPV-related BI at least one or more years prior to the interview, with the longest being over 28 years prior.

**Figure 1:**
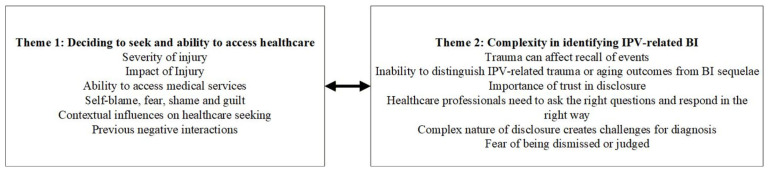
Themes and subthemes identified and discussed in this manuscript.

The analysis generated two overarching themes: ‘Deciding to seek and ability to access healthcare’, and ‘Complexity in identifying IPV-related BI’. Figure 1 shows subthemes supporting each theme which are described below. Participant characteristics are not provided alongside quotes to protect privacy and anonymity.

### 3.1. Theme 1: Deciding to Seek and Ability to Access Healthcare

Participants described a complex process involving many factors that either compelled or dissuaded them from accessing healthcare for potential BI following IPV (see Figure 1).

#### 3.1.1. Severity of Injury

Some participants described how having what they perceived as a severe injury gave them more confidence in being believed by health professionals if they disclosed what happened and would facilitate access to appropriate support and medical treatment.

*“It either gets so bad that you have no choice, you’re rushed to an ER like dying or that some sort of miracle just steps in and you’re able to get out. I can remember thinking, like almost wishing, that the injuries would have been worse, because then I felt like I wouldn’t be questioned. I wouldn’t be so much under the microscope. And like that’s a really crappy thing to feel and experience.”* (P012)

#### 3.1.2. Impact of Injuries

Participants described how persistent symptoms and impact on their ability to function increased their prioritization of seeking medical attention.

*“Being like in front of a screen or like a phone would hurt me, nauseous, definitely dizziness but I kinda ignored the symptoms at the beginning, so I didn’t go to the hospital until like couple of days after and that’s where they were like, oh, you’re like going through the end of the concussion.”* (P014)

#### 3.1.3. Ability to Access Medical Services

Even if the participant did perceive the need for medical help, it was not always easy for them to access healthcare services on their own. The partners/abusers often made it harder for women to seek help by removing access to resources they needed to book or attend a medical appointment.

*“I would have never made it to the to see a doctor. He would usually take my car keys and I mean, he had a set of car keys for my car and I had a set, but like I didn’t have any, you know, spare keys to his car, so like, he would always, you know, take my car or take my phone away. So I just was not in a position where I could go anywhere.”* (P008)

Consequently, participants described needing to come up with a plan to enable them to seek healthcare such as waiting for the abuser to go to work or pretending to travel elsewhere. In some cases, access was facilitated by friends, family, or work colleagues.

*“And only because I was at work after this incident and others told me that I need to go see a doctor because a lot of the times after these incidents I would be at home and my abuser would be quote unquote ‘taking care of me.’ So, this time, I was actually at work and people were, they were just like you need to go see a doctor.”* (P008)

#### 3.1.4. Self-Blame, Fear, Shame, and Guilt

In addition to the logistical challenges of seeking healthcare, women described experiencing complex emotional and psychological barriers which prevented them from seeking healthcare. This included fear of repercussion if the partner/abuser found out, self-blame, and/or feeling shame and guilt about what had happened.

*“The shelter I did go into, did suggest that I go see medical attention, but I was too scared to … as soon as they saw my neck they were like you need to go to the hospital girl and I was like, I can’t, I can’t, and so and so I didn’t go, and I should have. To this day I knew I should have gone, but I didn’t.”* (P011)

#### 3.1.5. Contextual Influences on Healthcare Seeking

While some participants were inclined to seek healthcare, contextual factors such as long wait times, cost, or distance to nearest healthcare center or other commitments (often, childcare-related) not specific to the IPV experience, ultimately made it difficult to access care from a healthcare professional.

*“I actually couldn’t go to the hospital because I have my kids, and so there was no way, like that particular time, I was 8 months pregnant with my youngest, and also had an infant in the house… and so there’s no way to navigate even getting ambulance transport. The hospital’s about 40 min from my house, with my kids on board of the ambulance,…but there was no getting there, getting back, so I couldn’t.”* (P012)

#### 3.1.6. Previous Negative Interactions

Some participants described those tending to them after IPV such as law enforcement not raising potential BI as in issue and in cases women felt dissuaded from seeking healthcare thinking it would not make a difference if they did. Additionally, there was a perception that healthcare professionals would not be able to do anything for their brain injury even if they did disclose what had happened to them.

*“I think that I ended up going to the hospital days after because I was feeling dizzy and very nauseous so I ended up going there. And then I was just telling them my symptoms at the moment and my symptoms prior and then I told them that I fell on my head. So they were like, oh, that makes sense. You probably have a concussion, but they didn’t really do like any like further testing. Like they touched my head where I had to see if I had any bumps and like I know I remember I had like a little bump over there. But that’s just what they said. Like they didn’t do anything.”* (P014)

### 3.2. Theme 2: Complexity in Identifying IPV-Related BI

Following the decision to seek healthcare, women talked about facing a dilemma: to enable health professionals to accurately assess them for potential brain injury, they needed to disclose the IPV and be able to describe what had happened. Six subthemes were identified within this theme. (See Figure 1).

#### 3.2.1. Trauma Can Affect Recall of Events

Participants described finding it hard to remember what had happened due to the trauma and confusion created by the perpetrator. This made it hard to describe the incident to healthcare professionals, which in turn made it harder for the health professionals to identify if there may have been a potential BI.

*“My eyes were burning. I couldn’t get out of bed, my head was heavy. So, this person was taking care of me during these times and I just was so confused and I couldn’t remember anything. It’s just so difficult to, cause I don’t even know how to explain any of these and I’m just like, where was I during this period of time? Because I can’t remember what happened in between, you know.”* (P008)

#### 3.2.2. Inability to Distinguish IPV-Related Trauma or Aging Outcomes from BI Sequelae

It was common for participants to talk about experiencing headaches, cognitive, neurological, and mental health difficulties that resulted following IPV and in some cases affected current or past functioning. However, participants expressed uncertainty about whether these difficulties were due to the injuries they sustained from being hit in the head, or because of stress and trauma they experienced from the IPV. Some even wondered if their symptoms were due to getting older. This difficulty in being able to untangle their symptoms and distinguish the root cause often made it difficult for participants to talk about their injuries with healthcare professionals at the time of injury.

*“Quantifying what changes happened because of that [TBI or concussion] versus just the just the other forms of abuse that I experience you know because like even today I still experience post trauma symptoms on occasion, it’s not regularly and it’s not frequently, but it does happen, I couldn’t tell you is it because of you know the concussion or TBI or is that because of the totality of the experience.”* (P002)

*“Yeah, I think it was like a mix of both, because I remember when I hit my head like I saw like flashes of lights like stars I guess and then I had like a bad headache afterwards, and was like pretty dazed, but I didn’t know if I was dazed because I hit my head, or because I was shocked at what happened because that was the first time I ever got hit.”* (P006)

#### 3.2.3. The Importance of Trust in Disclosure

Participants felt that seeking medical help from someone they knew and trusted enabled them to talk about their experiences. Many participants talked about disclosing their IPV to family doctors or primary care physicians, and were less comfortable disclosing to acute care doctors or specialists they did not regularly interact with. There was also concern that potential brain injuries could be used against them in court proceedings or affect custody of their children impacting on their disclosure and access to healthcare.

*“My [speciality type] doctor I did not tell him why I why this was going on, like I just was feeling this, and I was like, I need to be seen, and I thought at the time like I thought about asking him, like, you know, talking to him about that, but I just couldn’t bring myself to, I he was new, I didn’t know him that well, like, but my other doctors, my [other speciality type] … I knew him for a long time, so I did tell him like about it.”* (P004)

#### 3.2.4. Healthcare Professionals Need to Ask the Right Questions and Respond in the Right Way

The approach of healthcare professionals was often central in women’s decision-making about whether to disclose the IPV and describe what happened. Participants described that they needed their providers to ask questions regarding their safety or invite a conversation about the potential for IPV, to enable them to disclose.

*“When I told him I guess I must have fell and hit my eye or walked into something, you know, he [optometrist] was kind of like puzzled. You know had that puzzled look and stuff and and I just think he thought because of the impact, it was something different, you know, because it was like it was so red like, like the blood vessel was busted in my eye. And I think he knew, I think he knew something, ya. But you know he didn’t come out and say it.”* (P013)

Participants also described that, if questions were asked about potential IPV, it was not only the way the questions were asked but also the context (i.e., location, other people in the room) that either hindered or enabled them to disclose.

*“I went to…[the] hospital and in the emergency room. And they were trying to help me. But yeah, they’re asking me all these traumatic questions in front of this woman with this child, and I’m telling him like I don’t feel comfortable expressing myself in front of this child. This child needs not to be exposed to all of these traumatic questions. And I was very angry at the time from what had just happened. Yeah, I was still traumatized and I left.”* (P009)

#### 3.2.5. Complex Nature of Disclosure Creates Challenges for Diagnosis

For many participants, it became evident that it was very difficult to diagnose brain injury without having a clear understanding of what had happened. As a result, many women described common instances of misdiagnosis.

*“It wasn’t until they my primary doctor sent me to a neurologist for my migraines that we started looking into it. And then, You know, I’d never told anybody I’d been strangled or beat, or anything, and he wanted to start like testing me for MS because of the problems I was having…in that process, he asked me if I’d ever been hit in the head…he’s the first person who knew about it, and who was able to say, you sustained multiple brain injuries…he was the first, he was the person I told [about the abuse and repetitive head injuries]…it actually like was kind of relieving because it explained my symptoms, so then I can like focus on managing my symptoms.”* (P003)

#### 3.2.6. Fear of Being Dismissed or Judged

Several participants described situations where they felt dismissed by first responders or healthcare professionals because they were homeless or there was suspected drug and alcohol use, thereby navigating multiple potentially stigmatized identities. In many cases participants described being fearful that they would not be believed even if they did disclose both in healthcare and the judicial system. The fear of not being believed often arose because their partner/abuser had convinced them that they would not be taken seriously.

*“I think that they need to have these discussions like ‘they’re common and normal’ so that women, when women come in and talk about… I may have bumped my head. Well, ‘how did that injury take place?’ Woman’s not going to feel comfortable disclosing like hey, yeah, my partner did this to me unless she believes, understands that she’s not gonna get any bit of judgment from the person that they’re communicating with. The moment they feel they sense that they’re experiencing any kind of judgment, they’re going to shut down.”* (P002)

## 4. Discussion

Our qualitative findings present reasons why women who sustain brain injuries from intimate partner violence do not seek care at the time of injury and highlight potential facilitators to improve healthcare seeking following IPV-related BI. These reasons include psychological, physical, and logistical challenges that influence both their decision and ability to seek healthcare despite having sustained IPV-related BI, compounded by existing challenges with disclosing IPV more broadly.

One factor unique to IPV-related BI was the reality that women in our study found it difficult to distinguish between potential brain injury symptoms, trauma responses, and natural aging. The overlap in symptomology prevented women from recognizing either the presence or severity of BI and its associated symptoms both at the time of injury as well as later with persistent symptoms, particularly if symptoms were perceived to be more psychological than physical (i.e., headache, dizziness). Prior studies have shown this to be a barrier among clinicians caring for refugees with BI, and we extend this finding in our study from the perspective of women who experience IPV [25]. To address this, frontline practitioners at domestic violence shelters or other community agencies can provide reassurance that healthcare professionals are able to provide effective treatments, including addressing co-occurring symptoms, even if they cannot distinguish the cause of symptoms. Additionally, frontline professionals in emergency departments should provide screening in a private and safe location that will allow for women to divulge information about potential hits to the head or non-fatal strangulation.

Our research also found that severe and more visible injuries facilitated healthcare seeking as women felt that they would be more likely to be seen as “legitimately” injured and taken more seriously by co-workers, police, shelter staff, healthcare professionals, and even family members. Women with less identifiable injuries were less likely to avail healthcare resources, further underscoring the difficulty many participants faced internally justifying care for symptoms that felt “invisible.” Previous qualitative studies have articulated IPV-related brain injuries as more difficult to detect because they often become more obvious in later-onset behavioral symptoms rather than immediately observable physical injuries [26,27]. Given that many participants reported their symptoms being overlooked or dismissed by healthcare professionals, the invisible nature of the injury necessitates improved training for community and healthcare professionals alike to detect and recognize symptoms of BI regardless of the presence of physical signs or symptomology. This also aligns with existing calls to further refine domestic violence screening questions and procedures, including to better capture IPV-related BI [28].

Similarly to prior research, our findings reaffirmed the prevalence of internal factors like shame, guilt, and self-blame, often driven by sociocultural factors, as well as contextual barriers like long wait times or transportation barriers in dissuading women with IPV-related BIs from accessing care [29]. These internal and environmental barriers were compounded by household-level barriers as women’s partners also blocked access to help-seeking, including to hospitals, or constructed false narratives of the consequences in seeking care, instilling self-doubt and/or fear of negative repercussions in participants. These findings highlight the key role that first responders, witnesses, and the social networks around the person play in facilitating access to healthcare services, through reassurance and problem-solving logical challenges to access services. For example, police officers and paramedics should be on alert for and actively inquire about traumatic forces to the head and strangulation, and facilitate access to medical care if such events are endorsed. Careful documentation of such findings is also important to provide later credibility regardless of whether a women chooses to accept medical attention. At the same time, these findings highlight the need for system-level changes to facilitate care for everyone, including women who have experienced IPV. This will require an effort integrating healthcare, social services, and justice system agencies to routinely assess for both IPV and BI in a trauma-informed and non-judgmental manner that expresses the common nature (e.g., “This is something that many people experience.”) of such experiences.

Even for participants who did access care, identification of BIs was difficult because it involved disclosing the IPV. For example, several participants shared instances of misdiagnosis with other neuropsychiatric conditions before they disclosed their IPV to providers, which facilitated appropriate diagnosis and treatment. This underscores the need for improved IPV detection more broadly to facilitate access for IPV-related BI more specifically. One element of this is the necessity for time and private space to facilitate disclosure of IPV in a safe environment that will also allow for detection of a BI thereafter.

Past negative experiences with healthcare or law enforcement systems, especially for one woman in the current study who had experienced homelessness and was transgender, also dissuaded healthcare seeking, and contributed to feelings of helplessness or perceptions that nothing could be done even if they sought medical care. This necessitates the need for greater trauma-informed education and services across sectors, including but not limited to healthcare. Prior research has highlighted the lack of trauma-informed behavior in healthcare settings [30,31]. Given that IPV is a leading contributor to homelessness for women, a risk factor for substance use disorder, and prevalent among transgender people, our findings also suggest the need for existing IPV-specific education to include discussions about overlap with other social determinants of health and social identities [32,33,34]. More trauma-informed and inclusive healthcare systems, particularly Emergency Departments which act as entry points to care, would facilitate greater trust in healthcare systems and ultimately service utilization for women with IPV-related BIs [35]. Notably, participants in our study highlighted the critical role of trusted, longer term healthcare professionals like primary care physicians in facilitating disclosure, highlighting the need to consider interventions that involve healthcare professionals women see repeatedly, rather than specialists alone.

There are limitations to this study. First, our sample size was small, which limited the generalizability of these findings. Although we captured some (though very limited) experiences of populations often overlooked even within IPV literature, i.e., transgender women, we did not include the experiences of people identifying as male and non-binary individuals, non-English speaking participants, and others with other marginalized social identities who may have additional barriers or unique experiences. Future studies with larger and more diverse samples would help us understand if there are additional barriers or healthcare-seeking considerations that we did not identify here. Additionally, the interview approach is subject to the limitations of retrospective recall and the memory problems accompanying BI. Other work that may capture the experiences of both the people who experience IPV as well as others familiar with the situation may be helpful in this regard. Nevertheless, the study offers new insights to the current understanding of healthcare seeking among IPV survivors with IPV-related BI.

## 5. Conclusions

This qualitative study shares the lived experiences of women who have experienced IPV-related BI to inform efforts to facilitate prompt and appropriate treatment for IPV-related BI. These data reflect a range of barriers partially dependent upon the context of potential healthcare-seeking. For example, with respect to timeframe, acute barriers may be related to restricted or thwarted access from an abuser, whereas in the longer term, there may be challenges related to misattribution of symptoms (e.g., to trauma or aging). While some of these barriers are less amenable to change (e.g., physical barriers such as abusers preventing access to healthcare), many barriers require education and awareness raising about IPV to facilitate disclosure of abuse that resulted in a BI. General domestic violence education and training for women in general (regarding issues such as stigma), first responders, and healthcare professionals will be critical to making advancements in prompt and effective care for women who have experienced IPV-related BIs.

## Figures and Tables

**Table 1:** Demographics and brain injury characteristics.

Category	Number of Women (%)
**Race**	
White	10 (63)
Asian	1 (6)
Black	2 (13)
Bi/multi-racial	2 (13)
Other	1 (6)
**Ethnicity**	
Hispanic	3 (19)
Non-Hispanic	13 (81)
**Age**	
18–29	5 (31)
30–49	8 (50)
50–64	3 (19)
**Number of women sustaining IPV-related BI**	
TBI (blunt force trauma)	14 (88)
Strangulation-related AIC	11 (69)
**Number of TBIs for each woman**	
0	2 (13)
1–4	11 (69)
5–10	1 (6)
>10	2 (13)
**Number of Strangulation-related AICs for each woman**	
0	5 (31)
1–4	10 (63)
5–10	1 (6)
>10	0 (0)

Note: IPV = intimate partner violence; BI = brain injury; TBIs = traumatic brain injuries; AICs = alterations in consciousness.

## Data Availability

The data presented in this study are available on request from the corresponding author due to confidentiality reasons.

## Supplementary Materials

The following supporting information can be downloaded at: https://www.mdpi.com/article/10.3390/healthcare14020165/s1.

## Abbreviations

The following abbreviations are used in this manuscript:

IPV	Intimate Partner Violence
TBI	Traumatic Brain Injury
BI	Brain Injury
ER	Emergency Room
