# Supplementary material for: “I Can Remember Thinking, Like Almost Wishing, That the Injuries Would Have Been Worse, Because Then I Wouldn’t Be Questioned”: A Qualitative Study on Women’s Experience of Accessing Healthcare for Intimate Partner Violence-Related Brain Injury
Source: Healthcare (Basel). 2026 Jan 8;14(2):165. doi: 10.3390/healthcare14020165 (PMC12841078; doi:10.3390/healthcare14020165)
Supplement: Supplementary file 1 [file healthcare-14-00165-s001.zip › healthcare-3996470-supplementary.pdf]

Follow-up Qualitative Interview

Hello [name] thank you so much for agreeing to talk more with us about your past experiences. Before we start, I want to confirm that you are in a safe, private place that will be free from interruptions. Can you please confirm this is the case? Also, I want to ask your permission to audio and video record our meeting for data analysis purposes as we discussed previously. Although we will record this zoom session, we will immediately delete the video recording and only keep the audio recording so that we can use it for transcription purposes. Is it ok for us to audio and video record this meeting? *[Only proceed if subject confirms both safe location and recording. If either are not "ok," please either ask to reschedule or let her know that we can only proceed once and if she is ever comfortable with the recording.]*

This should take approximately 1 hour. As we did previously, today I will be asking questions about past physical partner violence which may be upsetting to some women. If this is the case for you and you need a break or have any questions at any time, please let me know. This will be much quicker than the previous study and there are no right or wrong answers. This is just about your personal beliefs and experiences. We want to understand these experiences from your perspective so that ultimately, women will be able to receive better help and treatment when necessary. If you are ready, we will get started. Are you ready? [If yes, proceed. If no, ascertain what the situation is and proceed accordingly.]

I want to start by re-asking some of the questions we asked when you participated previously but then allowing you to elaborate on those experiences and what they were like for you. So, some of this will feel familiar, but on this occasion, you will have more of an opportunity to share what you think is important about these experiences.

To start, after anything your partner did to you, did you ever:

- lose consciousness or black out?
- feel dazed or disoriented?
- have memory problems for what happened?

What happened? [ASKING WHAT PARTNER DID TO HER]

What was that like for you? [ASKING FOR HER EXPERIENCE OF BLACKING OUT, BEING DAZED ETC.]

After anything your partner did to you, did you ever:

- feel dizzy?
- see starts or spots?

What happened? [ASKING WHAT PARTNER DID TO HER]

What was that like for you? [ASKING FOR HER EXPERIENCE OF FEELING DIZZY, SEEING STARS AND SPOTS]

Understanding of TBI

What is your understanding of what a traumatic brain injury is?
What is your understanding of what a concussion is?

Did anyone ever tell you that you had a traumatic brain injury or concussion due to abuse from a partner?

[If so], who told you and why did they think you sustained one?
What was your response to this?
How did you feel after being told this?

Do you believe you ever sustained a traumatic brain injury or concussion because of something your partner did to you? Which one or both? How many times? [Approximations are fine if need be.]

<u>Perceived experience and consequences of IPV-related TBI</u>
Do you think a traumatic brain injury or concussion from a partner **has ever** caused difficulties with your physical, cognitive, or emotional health?
[If yes], what was the difficulty and when was this?

Do you think you are **currently experiencing** any difficulties related to a traumatic brain injury or concussion from a partner?
[If yes], what is the difficulty and when did it start?

<u>Experience and help seeking/offers following alterations of consciousness</u>

Did you ever **seek or consider seeking** medical treatment for [THE EXPERIENCE(S) SHE JUST REPORTED ABOVE]?
[If so,] why?
[If not], why not?
Were there specific barriers to seeking treatment?

Did you **seek or consider seeking help** from family, friends or community because you were worried about your brain?

Did you **seek or consider seeking help** from family, friends or community resources because of symptoms you thought might be related to [THE EXPERIENCE(S) SHE JUST REPORTED ABOVE]?
[If so,] why?
[If not], why not?
Were there specific barriers to seeking help from friends, family or community resources?

Was treatment ever **offered or received** for [THE EXPERIENCE(S) SHE JUST REPORTED ABOVE]? [If so], was the treatment helpful?
[If not], why not?
[If so,] what was or was not helpful about the treatment?

How did this experience affect how you thought, behaved or felt?

[IF STRANGLED WITH AN ALTERATIONIN CONSCIOUSNESS:] Did anyone look at your neck and talk to you about possible effects of being choked or strangled?

<u>How can this be done better?</u>
What could be done differently by health or support services to make it easier for people to get help after [THE EXPERIENCE(S) SHE JUST REPORTED ABOVE] from partner violence?

What services/resources would you want if you were to experience [THE EXPERIENCE(S) SHE JUST REPORTED ABOVE] from partner violence again?

What do you think can or should be done to make these services/resources accessible?

Is there anything else you would like to add?
